# Lipidomic Analysis of *Chlamydomonas reinhardtii* under Nitrogen and Sulfur Deprivation

**DOI:** 10.1371/journal.pone.0137948

**Published:** 2015-09-16

**Authors:** Dawei Yang, Donghui Song, Tobias Kind, Yan Ma, Jens Hoefkens, Oliver Fiehn

**Affiliations:** 1 Zhong Yuan Academy of Biological Medicine, Liaocheng People's Hospital/Affiliated Liaocheng Hospital, Taishan Medical University, 67 Dong Chang Xi Lu, Liaocheng, Shandong, 252000, P. R. China; 2 Department of Marine Science, College of Marine Science & Engineering, Tianjin University of Science & Technology 29, the 13th St., TEDA, Tianjin, 300457, P. R. China; 3 UC Davis Genome Center- Metabolomics, Davis, California 95616, United States of America; 4 King Abdulaziz University, Faculty of Science, Biochemistry Department, PO Box 80203, Jeddah 21589, Saudi Arabia; 5 Genedata Inc, Waltham, Massachusetts, United States of America; Louisiana State University Health Sciences Center, UNITED STATES

## Abstract

*Chlamydomonas reinhardtii* accumulates lipids under complete nutrient starvation conditions while overall growth in biomass stops. In order to better understand biochemical changes under nutrient deprivation that maintain production of algal biomass, we used a lipidomic assay for analyzing the temporal regulation of the composition of complex lipids in *C*. *reinhardtii* in response to nitrogen and sulfur deprivation. Using a chip-based nanoelectrospray direct infusion into an ion trap mass spectrometer, we measured a diversity of lipid species reported for *C*. *reinhardtii*, including PG phosphatidylglycerols, PI Phosphatidylinositols, MGDG monogalactosyldiacylglycerols, DGDG digalactosyldiacylglycerols, SQDG sulfoquinovosyldiacylglycerols, DGTS homoserine ether lipids and TAG triacylglycerols. Individual lipid species were annotated by matching mass precursors and MS/MS fragmentations to the in-house LipidBlast mass spectral database and MS2Analyzer. Multivariate statistics showed a clear impact on overall lipidomic phenotypes on both the temporal and the nutrition stress level. Homoserine-lipids were found up-regulated at late growth time points and higher cell density, while triacyclglycerols showed opposite regulation of unsaturated and saturated fatty acyl chains under nutritional deprivation.

## Introduction

Algae have been considered as promising third generation feedstocks for biofuel production. The advantages of algae use over terrestrial plants for biofuel generation include: algae do not compete with food crops, grow at high rates, and have higher oil yields exceeding that of conventional terrestrial plants. At the same time, algae can make use of industrial waste water to grow and reduce carbon dioxide emissions [1,2].

**Fig 1:**
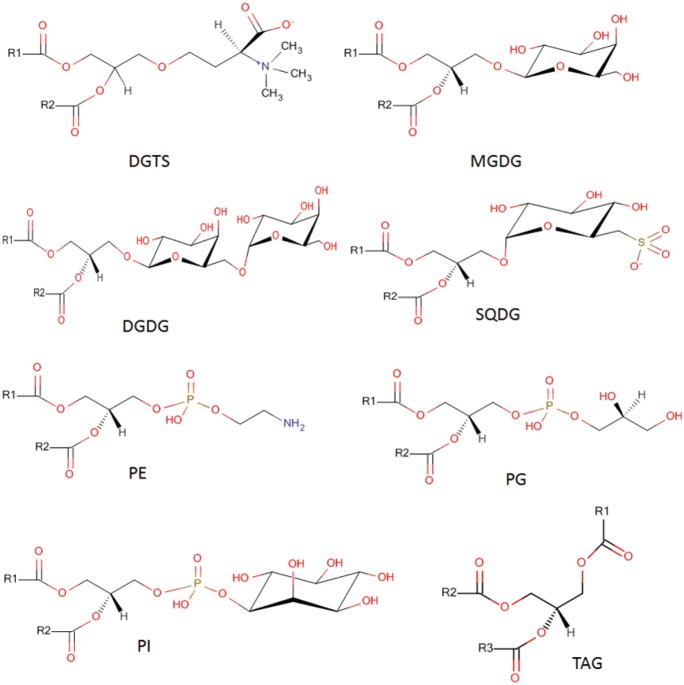
Common lipid species reported for *Chlamydomonas reinhardtii* cells. Labels R1, R2 and R3 represent different fatty acid acyl residues.

The single cell green algae *C*. *reinhardtii* serves as an important model organism for studying perturbations in metabolic pathways under environmental stress conditions [3–5]. Such stressors can include light and nutrients as well as temperature. The effect of nitrogen limitation on the lipid composition of *C*. *reinhardtii* has been studied [6–8]. When *C*. *reinhardtii* starved for nitrogen in stationary phase in the presence of exogenous acetate, those cells undergo a 15-fold increase in lipid body production within 48 h, and these lipid bodies consist of ～90% triacylglycerol and ～10% free fatty acid. A change of starch/lipid ratio with increased lipid production was observed under nitrogen deprivation conditions, even at a genetically starchless mutant *C*. *reinhardtii* [9]. RNA-seq and genetic analysis demonstrated that three acyltransferases, DGAT1, DGTT1, and PDAT1, have a role in triacylglycerol accumulation in *C*. *reinhardtii* under nitrogen starvation [10]. Sulfur, phosphorous, zinc and iron deficiency also resulted in increased lipid content in *C*. *reinhardtii* and other many algal species [11–15]. However, drastic and complete nitrogen deprivation also stops growth of algal biomass. A recent metabolic engineering report concluded that shunting carbon precursors from the starch synthesis pathway is more effective for increased triacylglycerol synthesis than a direct manipulation of lipid pathways [16]. Meanwhile, ambient temperature has a significant effect on the intracellular fatty acid of algae, such as *Chlorella vulgaris* and *Botryococcus*. *braunii*, but there was no effect on the content of acidic lipids sulfoquinovosyldiacylglycerols and phosphatidylglycerols in *C*. *reinhardtii* when temperature changed [17,18]. Light can also affect the lipid metabolism in algae. Typically, when algae grown at different light intensity, algae can be induced the formation of different kinds of lipids [19,20]. Most recently, it was shown that under partial nitrogen deprivation, biochemical remodeling of pathways enables *C*. *reinhardtii* cells to retain normal rates of cell division with a much more fine-tuned regulation of lipid biosynthesis [21]. This report had only analyzed the regulation of biosynthetic enzymes and primary metabolites [21], but not the effect of partial nutrient stress on the remodeling of complex lipids. We therefore now complement this study by comprehensively analyzing the relative composition of complex lipids in *C*. *reinhardtii* using shotgun lipidomics, a method that has been proven to be a powerful tool in global lipid analysis in a variety of species and organs[22,23]. Shotgun lipidomics using triplequadrupole mass spectrometry with direct infusion currently provides 158 annotated lipid species in plant extracts [24]. Such targeted methods are accurate, but might miss novel or unreported lipid species. Specifically, the lipid composition of *C*. *reinhardtii* had been studied with more classic tools such as thin-layer chromatography [25–28] and few studies with chromatography tandem mass spectrometry [8,27].Many lipid species were indentified including phosphatidylglycerols (PG), Phosphatidylethanolamines (PE), Phosphatidylinositols (PI), monogalactosyldiacylglycerols (MGDG), digalactosyldiacylglycerols (DGDG), sulfoquinovosyldiacylglycerols(SQDG),l,2-diacylglyceryl-3-O-4’-(N,N,N-trimethyl)-homoserine (DGTS) and triacylglycerols (TAG) (Fig 1). Most of previous studies usually focused on total lipid content, however, for a detailed interpretation of metabolic changes the molecular structures of lipids are needed when studying *C*. *reinhardtii* under different environmental perturbations.

## Material and Methods

### Culture growth and harvest of samples

Samples for lipids analysis were obtained from *C*. *reinhardtii* strain CC125 which was similar to previous published reports [29,30]. Briefly, the strain was cultivated in tris acetate phosphate (TAP) medium at 23°C under constant illumination with cool white fluorescent bulbs at a fluence rate of 70μmol m^−2^ s^−1^ and with continuous shaking. Cells were harvested by centrifugation, washed twice with sterile20 mM TRIS pH 7.0, supplied with 300 mM CaCl_2_, 400 mM MgCl_2_, and 7 mMKCl, and resuspended at a starting density of 2×10^6^ cells/mL in TRIS-buffered media under 3 different conditions (nitrogen deprivation: standard condition and subsequent decrease in ammonium acetate level: 75%, 50%of standard conditions; sulfur deprivation: standard condition and subsequent decrease sulfur level:75%, 50%of standard conditions).All cell numbers were counted using a hemacytometer and a microscope. Per time point studied, eight independent1ml samples were used in the nitrogen deprivation study, and six 1ml replicates were sampled during the sulfur deprivation study. Samples were harvested at 1h, 4h, 10h, 18h and 26h time points, respectively. At the incubation site, 1 mL cell suspensions were injected into 1mL of-70°C cold quenching solution composed of 70% methanol in water using a thermo block above dry ice. Pellets were flash frozen in liquid nitrogen and lyophilized at -50°C in 2 mL round bottom Eppendorf tubes.

### Lipid extraction

Lyophilized cells were disrupted using a MM 301 ball mill (Retsch GmbH & Co., Germany) for 3 minutes using a single 5 mmi.d. steel ball, followed by addition of 0.5 mL extraction solvent and vortexing for 10s and shaking at 4°C for 5min. Methanol:chloroform:water (MCW) (5:2:2) was used as the extraction solvent. Solvent ratios are given as volumetric measures. The solvent was degassed by directing a gentle stream of nitrogen through the solvent for 5 min. It was used prechilled to -20°C prior to extraction. After 2 min centrifugation at 16,100 rcf, the supernatants were removed followed by a secondary extraction step using an additional 800 μl extraction solvent, centrifugation and adding the supernatant to the first aliquot. Dried samples in a vacuum concentrator and kept at -80°C before further Nanomate-LTQ mass spectrometry analysis.

### Data acquisition and data processing

Before injection, the dried samples were re-suspended with 100μL methanol/chloroform (9:1) (degassed with nitrogen). The samples were vortexed and centrifuged for 2 min at 16,100 rcf. 10μL were taken out and diluted with 90uL methanol/chloroform (9:1) containing 7.5mM ammonium acetate. 20 ul sample volumes were pipetted into 96-well plates for analysis.

Mass spectrometric analysis was performed with an LTQ(Thermo Fisher Scientific, San Jose, CA)equipped with a Nanomate robotic nano-flow ion source (Advion, Ithaca, NY). The Nanomate cooling plate was set to 10°C, the Nanomate gas pressure to 0.4 psi and the voltage to 2.0 kV, and the source was controlled by the instrument’s Chipsoft 6.3.2 software. The samples were aspirated robotically from the 96-well plate and infused into the mass spectrometer through separate nozzles on an electrospray chip to avoid cross-contamination in comparison to conventional nanoelectrospray [31].The mass scan ranged from 350Da to 1100Da via positive and negative mode with a 60 s acquisition time. Afterwards, a data-dependent MS/MS method collected tandem mass spectra in positive and negative mode over a range of 10 minutes infusion time. In order to increase the number of MS/MS spectra for individual lipids, the m/z range in the method was split from 350–450 Da, 450–750 Da, 750–850 Da and 850–1100 Da. Lipid species were annotated using the in-house LipidBlast library consisting of over 200,000 lipid mass spectra [32]. A precursor window of 0.4 Da and a product ion search window of 0.8 Da was used. Scoring was performed using the NIST MS Search GUI with implemented dot product, reverse dot product and MS/MS probability matching. Hit scores of 999 presented optimal hits, hit scores lower than 400 were not considered. Lipid annotations were also performed using MS2Analyzer [33]. The MSMS data from both positive and negative modes were analyzed by MS2Analyzer including the calculated precursor ion masses, acyl side chain masses. All lipid annotations were manually verified. Infusion mass spectra were aligned using the Expressionist Refiner MS software (Genedata, Waltham, MA).Statistical evaluation was performed using the Statistica data miner package (Statsoft Tulsa, v9).

## Results and Discussion

### Mass spectral data processing and lipid annotation

We have used the Genedata Expressionist for MS software to find, quantify and align mass spectral ion traces even if masses slightly shifted during infusions (S1 Data). Results were compared to manual peak tracking and exporting using the mass spectrometer’s Xcalibur software for randomly chosen ions for different time points and stress conditions (S1 and S2 Figs). This direct comparison showed that the Expressionist for MS software correctly picked and aligned all peaks at a fraction of the time needed for manual analysis in the Xcalibur software. This automatic alignment procedure enabled processing hundreds of files comprising hundreds of ion traces within minutes of total processing time.

Next, we set out to annotate individual precursor ions by lipid structures. Classical algal lipid analysis involves transmethylation of complex lipids to fatty acid methyl ester (FAME) and quantification of the individual fatty acyl groups by flame ionization detection (FID) or mass spectrometry[34–36]. Although this method enables rapid overviews over fatty acyl contents in algal lipids extraction, it provides no information on the nature of intact lipids and potential differential regulation of specific lipid classes. Chip-based nanoelectrospray ionization tandem mass spectrometry has been widely used as lipid analysis tool [37,38], especially for high throughput lipidomics because the overall run times are in the range of one minute per sample, much faster than transmethylations and GC-FID analysis.

We have employed chip-based nanoelectrospray direct infusion coupled to iontrap utilizing data-dependent MS/MS scans in positive and negative mode to identify individual lipid species. Overall, more than 2,500 MS/MS precursors were collected in positive mode and around 1,000 MS/MS spectra were acquired in negative mode, rendering the complete annotation of all mass spectra by manual spectral interpretation impossible. Instead, we have aimed at using mass spectral matching to authentic lipids in analogy to approaches conducted in GC/MS. Searching public mass spectral databases, including MassBank (www.massbank.jp) with around 15,000 MS/MS spectra, the RIKEN MSn Spectral Database for Phytochemicals (http://spectra.psc.riken.jp/) with around 9,000 MS/MS spectra and Lipidmaps (http://www.lipidmaps.org/) [39] yielded only few potential hits. Instead, we have used an in-house library of mass spectra that is based on in-silico extension of lipid mass spectra by varying the acyl chain lengths and degree of double bonds of a range of authentic lipid reference standards [40,41]. This in house MS/MS library is called LipidBlast and contains more than 200,000 MS/MS spectra [32,41,42].MS/MS spectra were also screened for lipid-specific mass spectral features such as product ions and neutral losses using MS2Analyzer [33].

**Fig 2:**
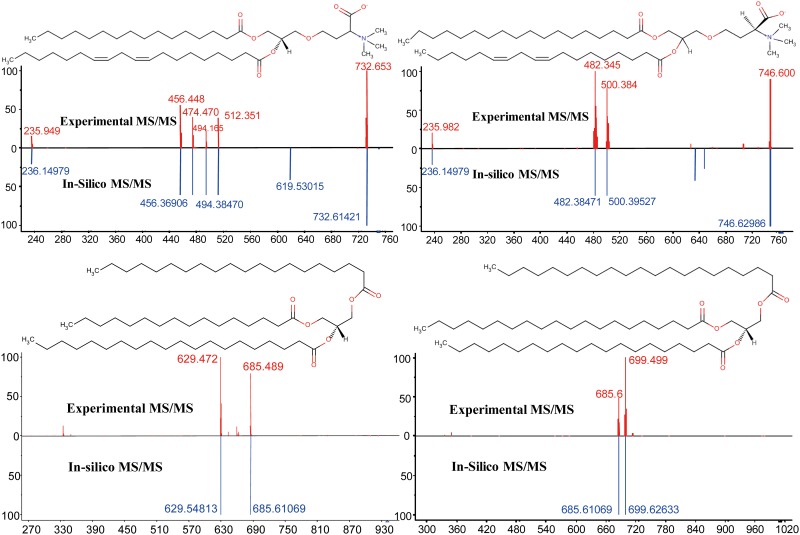
Annotation of complex lipids in algae by matching nanoelectrospray-linear ion trap MS/MS low resolution fragment spectra against the UC Davis LipidBlast library. Mass accuracy is <0.4 Da. Upper left panel: Annotation of the MS/MS spectrum from precursor m/z 750.9 Da as betaine lipid DGTS 35:2 (16:0/19:2); Upper right panel: Annotation of betaine lipid DGTS 36:2 (18:0/18:2); Lower left panel: Annotation of triacylglycerol TAG 56:0 (16:0/20:0/20:0); Lower right panel: Annotation of triacylglycerol TAG 61:0 (20:0/20:0/21:0).

When both MS2Analyzer and LipidBlast searches were combined, overall 60 lipids were unambiguously annotated in *C*. *reinhardtii* (Table 1).Among all the 60 annotated lipids, 27 lipids were annotated using both LipidBlast and MS2Analyzer. While 11 lipids were only annotated using LipidBlast queries and 22 lipids were only annotated usingMS2Analyzer. The low mass accuracy of the instrument and stringent use of high match scores may explain the low identification rates. Furthermore, isobaric interferences and ion suppression in direct infusion mode may lead to overlapping peaks and mixed-compound tandem mass spectra. However, the remaining identified compounds were annotated with high confidence. Fig 2 showed that the experimental MS/MS spectra had good dot product matches with Lipidblast MS/MS library. All head groups and acyls were confirmed by MS2Analyzer. Most of the lipids commonly described for *C*. *Reinhardtii* (Fig 1) were positively identified in this manner, including DGTS, MGDG, DGDG, SQDG and TAG (Table 1). Among them, PG, PI, and SQDG were detected in negative mode. MGDG, DGDG and SQDG are major components of photosynthetic membranes which account for around 70% of total membrane in *C*. *reinhardtii* [27]. Extraplastidial membranes of *C*. *reinhardtii* do not contain phosphatidylcholine lipids, but instead comprise of the non-phosphorous betaine lipid DGTS [25,26]. DGTS substitutes for phosphatidylcholines (PC) as a major membrane component that is discussed to fulfill similar functions for the overall membrane structure as PCs perform in other organisms [43]. Using MS/MS analysis via positive ionization mode, betaine lipids are easily annotated by their dominant product ion m/z 236 [41,44]. Neutral loss analysis from the precursor ions accounted for the enumeration of different fatty acid acyl chains to identify individual DGTS species. For example, a precursor750.76 Da was detected as precursor for an experimental MS/MS spectrum which matched very well the LipidBlast MS/MS spectrum of DGTS (16:0/19:2) and its precursor ion [M+H]^+^m/z750.625. In order to validate this LipidBlast match, we performed a manual spectral interpretation of the experimental MS/MS spectrum. The experimental MS/MS fragment ion m/z 732.6 represented a water loss from the precursor ion; m/z 512.4 and m/z 494.2represented a neutral loss of a palmitoyl acyl chain (256.7 Da) from the intact precursor ion and its water loss fragment, respectively; fragment ions m/z 474.5 and 456.4the loss of the odd-chain nonadecanoyl group with two unsaturated bonds (294.4 Da; C19:2) and finally, m/z 235.9 represented the residual mass of the DGTS backbone and head group after the loss of both fatty acid acyl chains. We found this mass spectral interpretation in clear agreement with the automatic Lipidblast and MS2Analyzer annotation. However, MS/MS analysis alone does not enable assigning accurate stereochemical and regiospecific positional isomers; hence, final assignments of sn1/sn2 positions and the correct positioning of the unsaturated double bonds is not possible without using further techniques.

**Table 1:** Annotated lipids in *C*. *reinhardtii* under nitrogen and sulfur stress conditions. The reverse dot product represents the level of confidence from in silico-MS/MS library search. Compound annotations without reverse dot product were annotated using MS2Analyzer.

Experimental mass m/z	Precursor*m/z* library	Rev-Dot library	Adduct	Annotated Species
741.471	741.683	516	[M-H]-	PG 34:4 (16:1/18:3)^c^
743.685	743.486	502	[M-H]-	PG 34:3 (16:1/18:2)^c^
745.50	745.58	650	[M-H]-	PG 34:2 (16:1/18:1)^c^
793.69	793.73	NA	[M-H]-	SQDG 32:0(C16:0/C16:0)^b^
815.68	815.74	NA	[M-H]-	SQDG 34:3(C18:3/C16:0)^b^
817.68	817.58	NA	[M-H]-	SQDG 34:2(C18:2/C16:0)^b^
819.72	819.77	NA	[M-H]-	SQDG 34:1(C18:1/C16:0)^b^
835.75	835.534	NA	[M-H]-	PI 34:1 (16:0/18:1)^c^
474.711	474.379	NA	[M+H]+	LysoDGTS 16:0^b^
496.740	496.364	NA	[M+H]+	LysoDGTS 18:3^b^
680.451	680.546	673	[M+H]+	DGTS 30:2 (14:2/16:0)^c^
704.882	704.546	868	[M+H]+	DGTS 32:4(16:0/16:4)^c^
706.91	706.562	915	[M+H]+	DGTS 32:3 (16:0/16:3)^c^
708.51	708.578	899	[M+H]+	DGTS 32:2 (16:0/16:2)^c^
732.37	732.578	900	[M+H]+	DGTS 34:4 (16:0/18:4)^c^
734.41	734.593	756	[M+H]+	DGTS 34:3 (16:0/18:3)^c^
736.32	736.609	773	[M+H]+	DGTS 34:2 (16:0/18:2)^c^
738.407	738.625	762	[M+H]+	DGTS 34:1 (16:0/18:1)^c^
748.90	748.609	869	[M+H]+	DGTS 35:3(16:0/19:3)^c^
750.76	750.625	880	[M+H]+	DGTS 35:2 (16:0/19:2)^c^
752.44	752.640	900	[M+H]+	DGTS 35:1 (16:0/19:1)^c^
754.56	754.5622	782	[M+H]+	DGTS 36:7 (18:3/18:4)^c^
756.20	756.578	857	[M+H]+	DGTS 36:6(18:3/18:3)^c^
758.68	758.5935	911	[M+H]+	DGTS 36:5(18:2/18:3)^c^
760.74	760.609	765	[M+H]+	DGTS 36:4 (18:1/18:3)^c^
760.600	760.609	NA	[M+H]+	DGTS 36:4 (18:2/18:2)^b^
762.23	762.625	796	[M+H]+	DGTS 36:3 (18:1/18:2)^c^
762.550	762.625	NA	[M+H]+	DGTS 36:3 (18:0/18:3)^b^
764.866	764.640	784	[M+H]+	DGTS 36:2 (18:1/18:1)^c^
764.29	764.640	896	[M+H]+	DGTS 36:2 (18:0/18:2)^a^
772.922	772.609	900	[M+H]+	DGTS 37:5 (18:3/19:2)^a^
774.939	774.625	874	[M+H]+	DGTS 37:4 (18:3/19:1)^a^
776.963	776.6404	863	[M+H]+	DGTS 37:3 (16:0/21:3)^a^
786.762	786.6248	903	[M+H]+	DGTS 38:5 (18:3/20:2)^a^
788.906	788.6404	911	[M+H]+	DGTS 38:4 (18:3/20:1)^a^
802.845	802.6561	848	[M+H]+	DGTS 39:4 (18:1/21:3)^a^
762.47	762.516	NA	[M+H]+	MGDG 34:7(16:4/18:3)^b^
798.42	798.609	NA	[M+H]+	MGDG 36:3(16:0/20:3)^b^
799.8028	799.53	999	[M+Na]+	MGDG 36:5 (18:2/18:3)^a^
929.801	929.524	NA	[M+Na]+	DGDG 34:7(16:3/18:4)^b^
931.23	931.539	NA	[M+Na]+	DGDG 34:6(16:3/18:3)^b^
936.95	936.662	NA	[M+NH4]+	DGDG 34:1(16:0/18:1)^b^
937.20	937.5865	869	[M+Na]+	DGDG 34:3 (16:0/18:3)^c^
939.83	939.608	810	[M+Na]+	DGDG 34:2 (16:0/18:2)^c^
818.030	817.632	NA	[M+Na]+	TAG 48:6(16:2/16:2/16:2)^b^
868.688	868.739	NA	[M+NH4]+	TAG 52:6(16:0/18:2/18:4)^b^
866.725	866.818	NA	[M+NH4]+	TAG 52:7(16:0/18:3/18:4)^b^
868.517	868.739	914	[M+NH4]+	TAG 52:6(16:0/18:3/18:3)^c^
941.804	941.97	800	[M+Na]+	TAG 56:0(16:0/20:0/20:0)^c^
955.830	955.773	NA	[M+Na]+	TAG 58:7(16:0/20:1/22:6)^b^
957.820	957.789	NA	[M+Na]+	TAG 58:6(16:0/20:1/22:5)^b^
959.910	959.804	NA	[M+Na]+	TAG 58:5(16:0/20:0/22:5)^b^
958.8274	958.93	948	[M+NH4]+	TAG 58:3(18:2/20:0/20:1)^a^
970.00	969.883	NA	[M+Na]+	TAG 58:0(18:0/20:0/20:0)^b^
983.8795	983.90	991	[M+Na]+	TAG 59:0(19:0/20:0/20:0)^a^
986.8871	986.94	901	[M+NH4]+	TAG 60:3(18:1/20:1/22:1)^c^
997.8779	997.91	999	[M+Na]+	TAG 60:0(20:0/20:0/20:0)^c^
1011.834	1011.93	984	[M+Na]+	TAG 61:0(20:0/20:0/21:0)^a^
1013.549	1013.851	NA	[M+Na]+	TAG 62:6(20:0/20:0/22:6)^b^
1015.980	1015.867	NA	[M+Na]+	TAG 62:5(20:0/20:0/22:5)^b^

^a^: represented these lipids only can be annotated using Lipidblast;

^b^: represented these lipids only can be annotated using MS2Analyzer;

^c^: represented these lipids can be annotated by both databases.

Thylakoid lipids in most vascular plants and algae are synthesized either by the chloroplast (prokaryotic pathway) or by the endoplasmic reticulum (eukaryotic pathway) [45]. However, unlike in higher plants, *C*. *reinhardtii* employs its own autonomous biosynthetic pathway by assembling galactoglycero lipids in the chloroplast. Therefore, MGDG, DGDG and SQDG in *C*. *reinhardtii* contain exclusively C16 fatty acids at the *sn*-2position of the glycerol backbone [25,46]. Correspondingly, SQDG and DGDG lipids are all presented with palmitoyl residues in the *sn*-2position (Table 1). We found the dipalmitolyl lipid SQDG (16:0/16:0) as predominant SQDG in *C*. *reinhardtii* in accordance to previously published results [25]. DGTS lipids should contain mostly octadecanoyl fatty acids in the *sn*-2 position [25]. Our study demonstrated that some DGTS lipids may also comprise C19 and C20 fatty acids in the *sn*-2 position, while we confirmed that most of DGTS lipids indeed had C18-residues in the *sn*-2 position. More surprisingly, we detected clear evidence for odd-chain fatty acyl groups (C17 and C21)in neutral lipids, triacylglycerols. TAGs with C19 and C17 acyl chains were founded in *C*. *reinhardti* [8].We did not observe PE that were reportedly detected by thin layer chromatograph (TLC) in *C*. *reinhardtii* [25,26,28]. Vieler et al [27] reported that PE constitute less than 5% of the total content of complex lipids in *C*. *reinhardtii*. It is possible that such minor components might have remained undetected in our direct infusion approach, for example due to isobaric interferences.

### Effect of nitrogen and sulfur deprivation on growth rates and lipidomic phenotypes of *C*. *reinhardtii*

**Fig 3:**
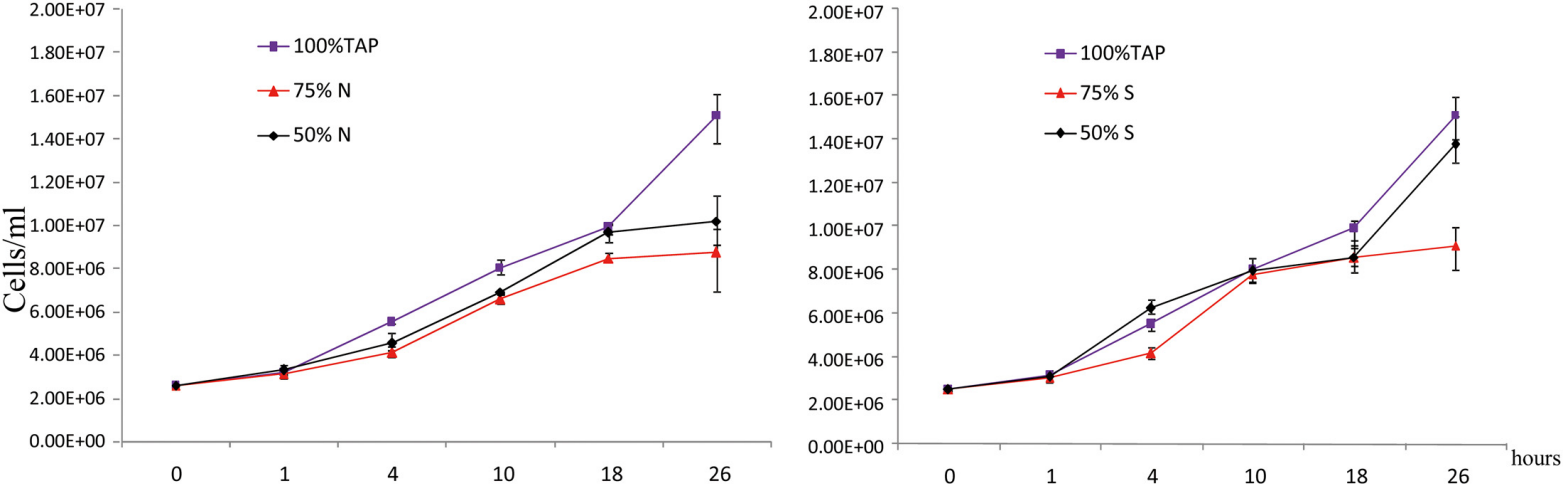
Growth curves of *C*. *reinhardtii* after transfer to nitrogen-deprived (left panel) or sulfur-deprived media (right panel). The values are averages ±SE (standard deviation) for six replicate culture flasks.

Growth curves of *C*. *reinhardtii* CC125 showed that cell growth rates were unaffected by N-levels of 75% or 50% of normal condition (TAP, 100%N) for at least 18 hours, about 3 cell cycles (Fig 3). The same results were obtained for *C*. *reinhardtii* CC125 grown under different sulfur levels in comparison to normal sulfur levels. However, cell growth would be slightly different after 18h and cell numbers would grow to 1.5×10^7^/ml when *C*. *reinhardtii* grows under normal condition. Inversely, *C*. *reinhardtii* growing under nutrient deprivation media turned to grow slowly after 18h compared to TAP media. We have not completely starved cultures of nitrogen or sulfur supply, as it is well known that cell division is halted when *C*. *reinhardtii* cultures are depleted of nitrogen containing media[21,35]. In contrast, in our experimental design we studied the modulation of lipid composition under stress conditions under which algal cells were still alive and actively dividing.

**Fig 4:**
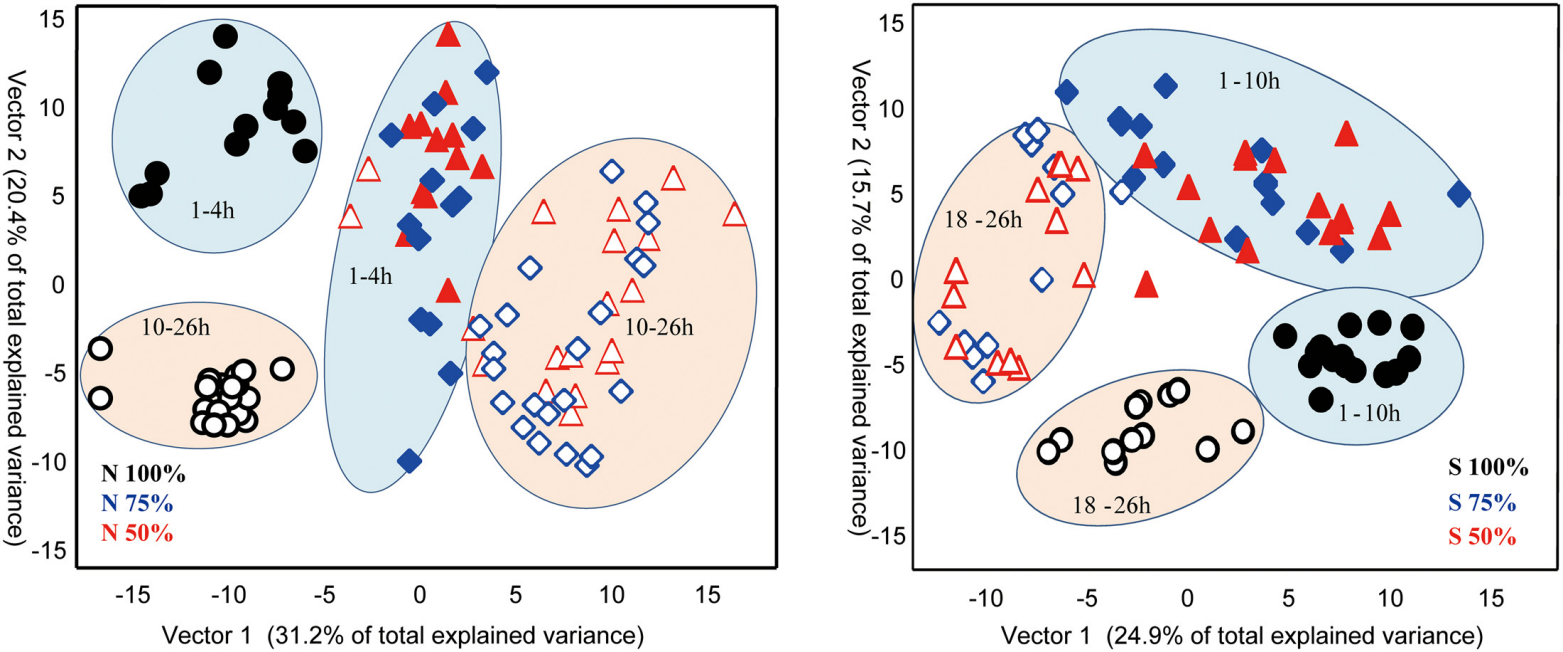
Partial Least Square supervised multivariate analysis of lipids under nutrient deprivation conditions at time points ranging from 1h, 4h, 10h, 18h and 26h. Closed symbols reflect samples taken at early exponential growth rates, open symbols denote samples harvested at late growth stages. Left panel: Lipidomic phenotypes of *C*. *reinhardtii* cells grown under normal nitrogen-containing media (TAP, 100%N) or under reduced nitrogen conditions (N75%, blue, and N50%, red). Right panel: Lipidomic phenotypes of *C*. *reinhardtii* cells grown under normal sulfur-containing media (TAP, 100%S) or under reduced sulfur conditions (S75%, blue, and S50%, red).

Overall profiles clearly showed the effect of deprivation of both nitrogen and sulfur contents in the media (S3 Fig). Unsupervised Principal Component Analysis (PCA) readily distinguished the lipidomic profiles under normal growth conditions from any of the two stress conditions. Under nitrogen deprivation, an additional clear separation of profiles of early time points and late growth time points were observed. For sulfur deprivation, unrelated variance in the data set was found to be higher than for the nitrogen experiment, and only vectors 2 and 3 (that explained less amount of the total variance than vector 1) were related to parameters of the study design and separated the 100% complete sulfur conditions from the 75% and 50% sulfur-depleted growth media. In order to get clearer lipidomic phenotype clusters we performed supervised Partial Least Square multivariate regression analysis (PLS) by ignoring variance in the data set that was unrelated to either growth media conditions or growth time points. PLS score plots more readily visualized the extent of lipidomic differences between the growth conditions and time points (Fig 4). For both nitrogen and sulfur deprivation, lipidomic phenotypes were found to be drastically different from normal TAP media growth. Similarly, for both stress conditions the partly reduced nutrient content (75%) was indistinguishable from the more drastically reduced nutrient content (50%). On top of the differentiation of lipid clusters under nutrient stress, the PLS graphs (Fig 4) also clearly show temporal differences in the composition of complex lipids in *C*. *reinhardii* between early-growth and late-growth time points. This temporal pattern was found to be more pronounced and faster for nitrogen stress conditions than for reduced sulfur contents, reflecting the fact that many complex lipids comprise nitrogen in their structure which might lead to earlier remodeling in overall lipid compositions.

Besides the fact of overall modulation of lipid compositions, it is important to individually assess metabolic trends in different lipid classes under nutrient stress. Nitrogen is the most critical growth-limiting nutrient in photosynthetic organisms. The effect of nitrogen limitation on the fatty acid composition has been studied in *C*. *reinhardtii* wild-type and starch-less mutant, BAF-J5 [35]. It was found that the total fatty acids increased in wild-type and mutants, and the mutants produced significant levels of 16:0, 18:1 (9), 18:2 (9,12) and 18:3 (9,12,15) and low levels of long chain fatty acids under nitrogen deprivation [35]. Under nitrogen limitation condition, many algal species including *C*. *reinhardtii* can accumulate neutral lipids, mainly in the form of TAG, as a storage of energy and carbon in response to stress conditions [47]. Using thin layer chromatography, the SQDG, DGTS and PE lipids remained largely unaltered after nitrogen withdrawal [46]. However, there were no reports about the regulation of individual lipid species in algae under nitrogen deprivation condition.

**Fig 5:**
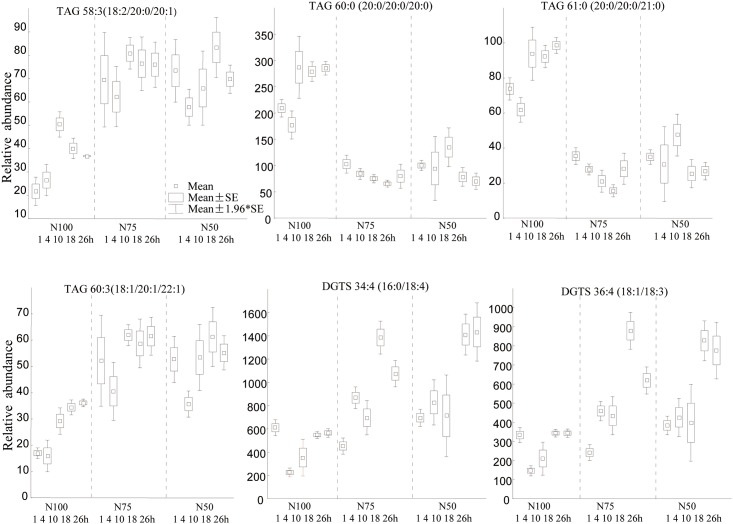
Univariate box-whisker plots of triacylglycerol and betaine lipid species in *C*. *reinhardtii* in temporal response to nitrogen deprivation. Arithmetic mean values with ±S.E. as box and ±1.96 S.E. as whiskers.

As demonstrated in Fig 5, we observed neutral lipids with a high degree of unsaturation, specifically TAG58:3 (18:2/20:0/20:1) and TAG 60:3 (18:1/20:1/22:1), to be increased under reduced nitrogen conditions compared to normal media-TAP (100% N). These findings were in agreement with previous studies reporting that *C*. *reinhardtii* accumulates neutral lipids under acute nitrogen starvation conditions [6,7]. Conversely, we found saturated triacylglycerols, specifically TAG 60:0 (20:0/20:0/20:0) and TAG 61:0 (20:0/20:0/21:0) to be significantly down-regulated under nitrogen deprivation conditions (Fig 5). This finding suggests differential activities of lipid desaturases in *C*. *reinhardtii* under nitrogen stress which might yield more fluid and permeable membranes. A substantiation of this novel hypothesis requires accurate quantification of more triacylglycerol species and detailed enzymatic studies.

DGTS homoserine ether lipids are very important for *C*. *reinhardtii*. This lipid class has been suggested to act as a substitute for phosphatidylcholines. DGTS 36:4 (18:1/18:3) and DGTS 34:4 (16:0/18:4) were significantly increased under nitrogen deprivation, especially at late exponential growth time points (Fig 5). Similar trends were observed for DGTS 36:3 (18:1/18:2), DGTS 36:2 (18:1/18:1), DGTS 34:3 (16:0/18:3), DGTS 34:2 (16:0/18:2), DGTS 34:1 (16:0/18:1) and DGTS 34:0 (16:0/18:0) (S4 Fig). However, DGTS 39:4 (18:1/21:3) was found decreased under nitrogen deprivation conditions and other homoserine lipids remained unaltered, specifically DGTS 35:3 (16:0/19:3) and DGTS 35:2 (16:0/19:2). A previous study showed that the amount of DGTS remained largely unaltered at 48h after nitrogen withdrawal [46]. Our study demonstrates a more nuanced view on DGTS metabolism. It appears that while total DGTS contents may not be altered under nitrogen stress conditions, there is a differential remodeling of even-chain DGTS in opposite to DGTS species that comprised odd-chain fatty acyl groups. In addition, a range of DGTS lipids showed a clear temporal regulation even under nitrogen replete conditions.

**Fig 6:**
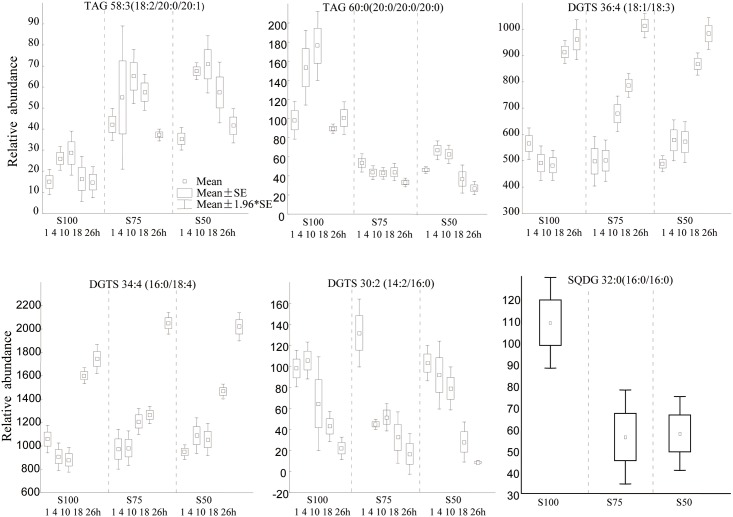
Univariate box-whisker plots of triacylglycerol, betaine and sulfoquinovosyl lipid species in *C*. *reinhardtii* in temporal response to sulfur deprivation. Arithmetic mean values with ±S.E. as box and ±1.96 S.E. as whiskers.

Sulfur (S), is a further macro-nutritional element critical for algal growth. Its effect on the acidic lipids in thylakoid membranes has been studied in *C*. *reinhardtii* [13,48,49]. We found the sulfolipid SQDG 32:0 (16:0/16:0) to be decreased under sulfur-deprived conditions relative to normal TAP media (Fig 6). This finding is in accordance with previous studies demonstrating that sulfur depletion can cause degradation of SQDG chloroplast membrane lipids in *C*. *reinhardtii* [48,49]. SQDG was also found to be degraded in order to supply sulfur for the synthesis of proteins as early as 6 h after sulfur withdrawal[48].Triacylglycerol regulation showed similar trends under sulfur stress as under nitrogen deprivation. Specifically, the highly desaturated TAG 58:3 (18:2/20:0/20:1) increased in 75%S and 50%S media compared to normal media, whereas the completely saturated TAG 60:0(20:0/20:0/20:0)decreased under sulfur stress. This finding shows that the potential difference in desaturase activities may be a generic stress response, rather than specific to the lack of a certain nutrient.

When *C*. *reinhardtii* exposed to sulfur deprivation, DGTS homoserine lipids did not show significant changes to the stress conditions (Fig 6). However, some DGTS lipids showed clear temporal changes along the growth curve. For the homoserine lipids DGTS 36:4 (18:1/18:3) and DGTS 34:4 (16:0/16:4), relative contents increased over time whereas DGTS (14:2/16:0) contents decreased almost linearly (Fig 6). Interestingly, there were no changes in DGTS 35:3 (16:0/19:3), DGTS 35:2 (16:0/19:2) and DGTS 35:1 (16:0/19:1) at different time points under sulfur deprived or normal condition (S5 Fig). The observed temporal trends of DGTS lipids were also found as high-impact metabolites driving the differentiation of overall lipidomic phenotypes in the PLS graphs between early stage (1-10h) and late stage (18-26h) growth (Fig 3). We suggest that DGTS lipids, constituting a major component of algal membrane, remodels in a temporal manner in response to overall cell density in addition to nuanced remodeling of odd-chain and even-chain lipids under nitrogen stress conditions.

## Conclusion

We have shown that chip-based nanoelectrospray direct infusion coupled to iontrap mass spectrometry can rapidly profile lipid extracts in algal extracts, specifically demonstrated for *C*. *reinhardtii*. Identification of major lipid species by tandem mass spectral fragment analysis concurred with findings reported by much more laborious thin layer chromatography/GC-FID analysis methods. Despite the caveats of relative quantification and the potential effects of ion suppression, multivariate and univariate analyses clearly showed that nanoelectrospary-MS lipidomic assays can directly be used for analyzing overall trends in lipid remodeling, including the extent and temporal basis of lipid regulation. Importantly, we demonstrated that under nutrient deprivation, unlike under complete nutrient starvation, lipid remodeling occurs in a specific manner for different lipid classes, different degree of desaturation level of acyl groups and different impact on odd-chain versus even-chain lipids. We suggest this tool to be easily used for high throughput screening of algal strains in biotechnology and biofuel production.

## Supporting Information

S1 Data Data file for lipidomic data, mass spectra metadata. Supplementary data set lists annotated lipids and all mass spectra under nutrient deprivation conditions at different time points.
(XLSX)

S1 Fig Evaluation of alignment results from direct infusion mass spectrometry experiments comparing Genedata’s Expressionist Refiner MS software to ThermoFisher’s instrument software Xcalibur for M/Z 734.91. (TIF)

S2 Fig Evaluation of alignment results from direct infusion mass spectrometry experiments comparing Genedata’s Expressionist Refiner MS software to ThermoFisher’s instrument software Xcalibur for M/Z 1011.83. (TIF)

S3 Fig Unsupervised Principal Component Analysis clustering lipidomic profiles under sulfur deprivation (left panel) and nitrogen deprivation (right panel). Black = TAP normal medium, blue labels: 25% reduction in nutritional input (N or S), red labels: 50% reduction in nutritional input in media (N or S).
(TIF)

S4 Fig Univariate box-whisker plots of individual homoserine (betaine) lipid species in *C*. *reinhardtii* in temporal response to nitrogen deprivation. Arithmetic mean values with ±S.E. as box and ±1.96 S.E. as whiskers.
(TIF)

S5 Fig Univariate box-whisker plots of individual homoserine (betaine) lipid species in *C*. *reinhardtii* in temporal response to sulfur deprivation. Arithmetic mean values with ±S.E. as box and ±1.96 S.E. as whiskers.
(TIF)
