# Supplementary figures and images for: Lipidomic Analysis of Chlamydomonas reinhardtii under Nitrogen and Sulfur Deprivation
Source: PLoS One. 2015 Sep 16;10(9):e0137948. doi: 10.1371/journal.pone.0137948 (PMC4574153; doi:10.1371/journal.pone.0137948)

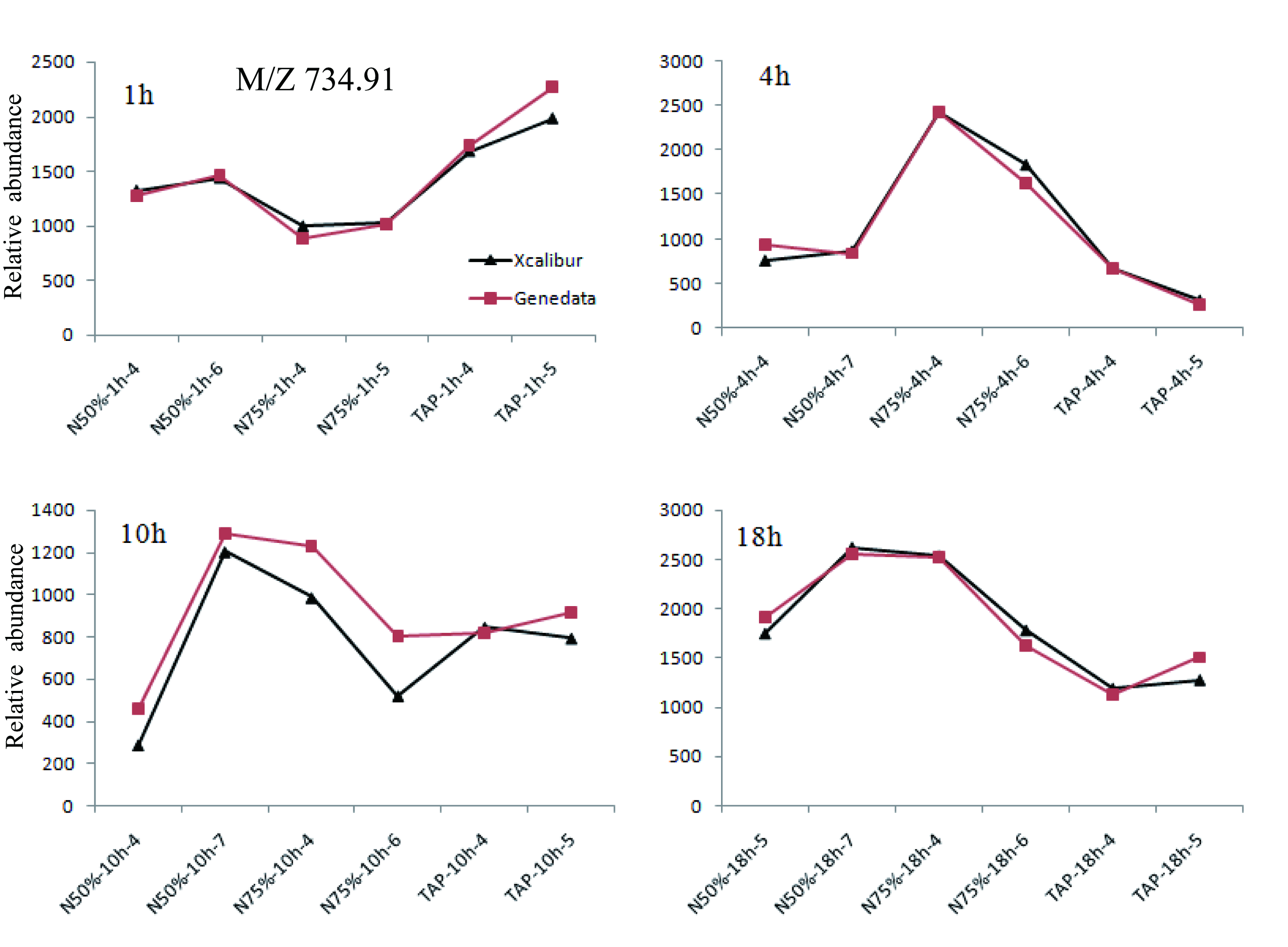

Supplement: S1 Fig — (TIF) [file pone.0137948.s002.tif]

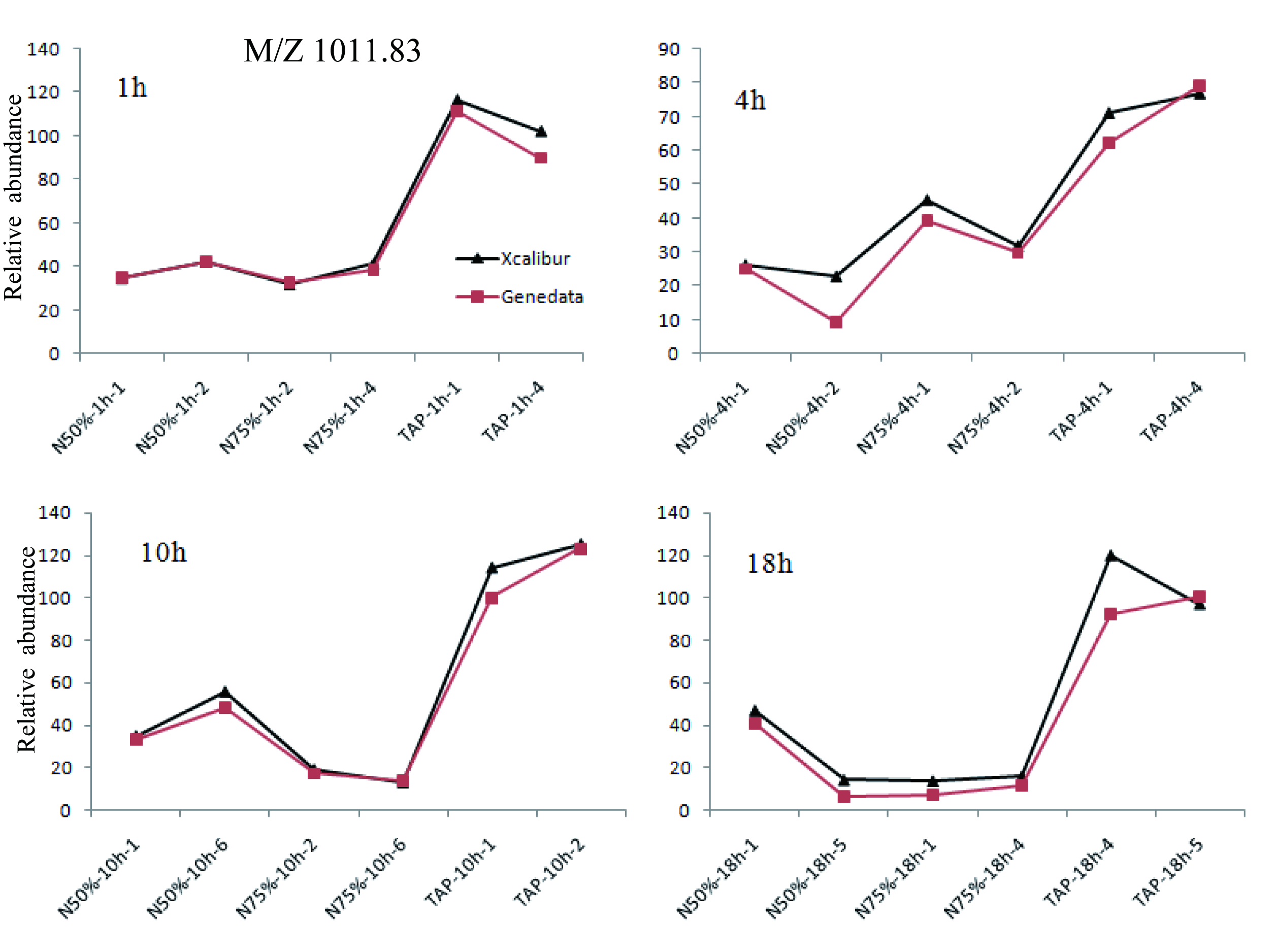

Supplement: S2 Fig — (TIF) [file pone.0137948.s003.tif]

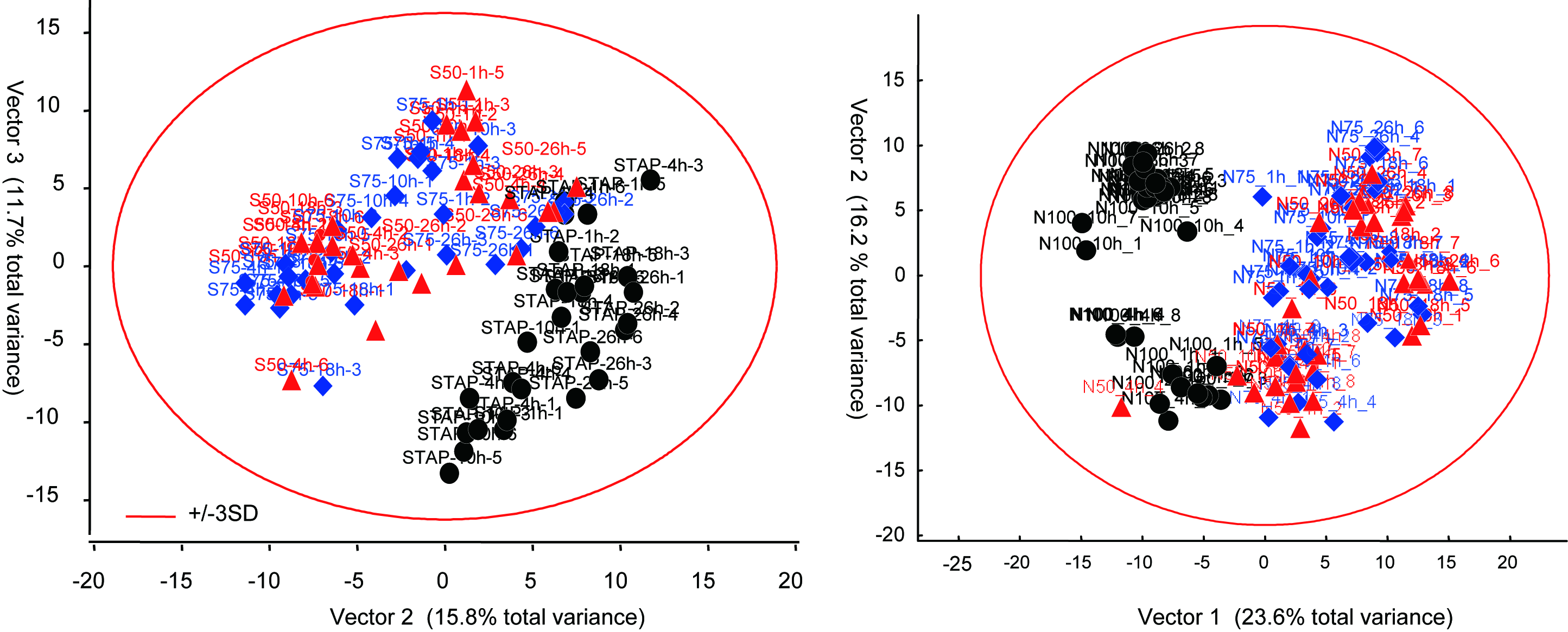

Supplement: S3 Fig — Black = TAP normal medium, blue labels: 25% reduction in nutritional input (N or S), red labels: 50% reduction in nutritional input in media (N or S). (TIF) [file pone.0137948.s004.tif]

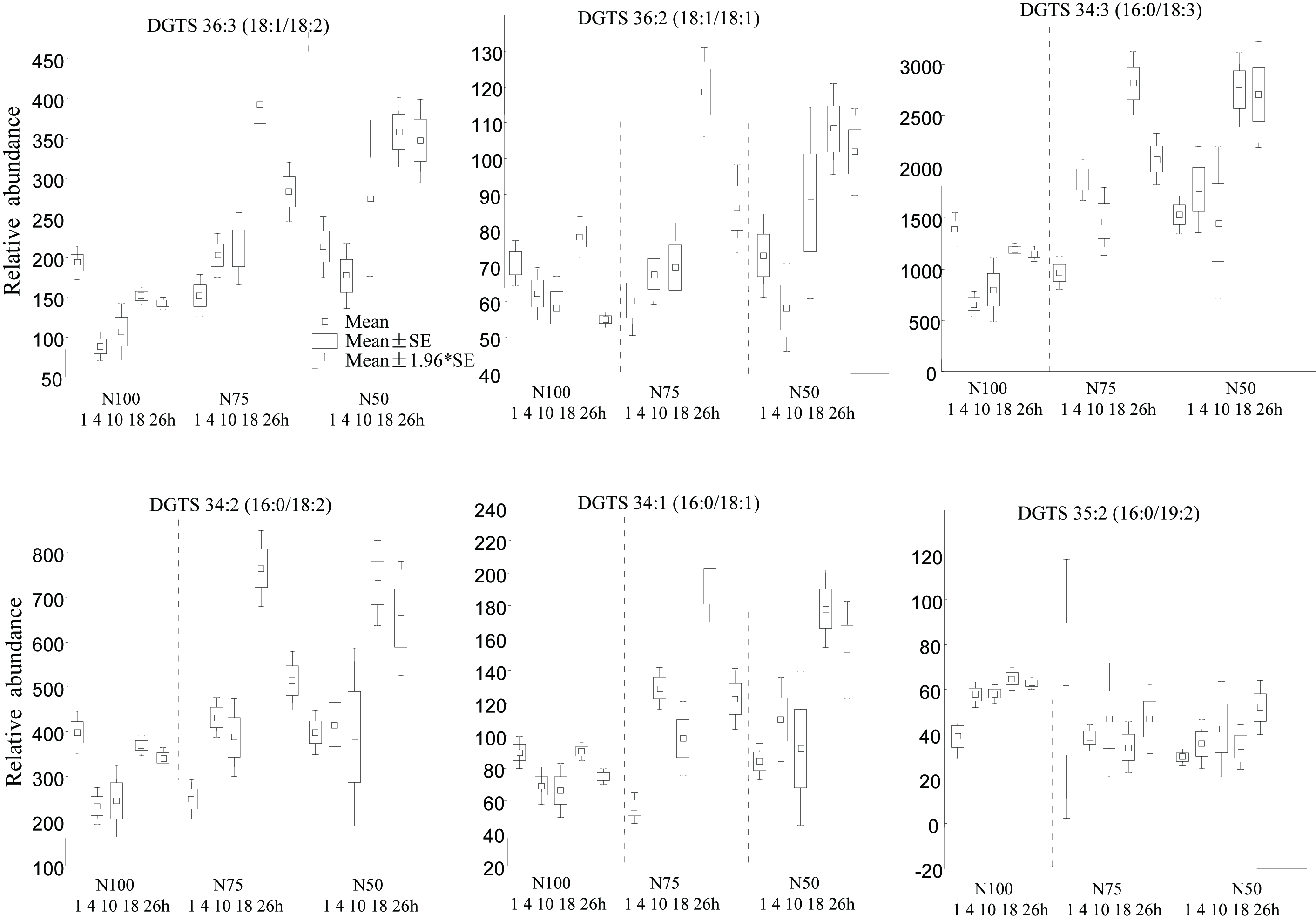

Supplement: S4 Fig — Arithmetic mean values with ±S.E. as box and ±1.96 S.E. as whiskers. (TIF) [file pone.0137948.s005.tif]

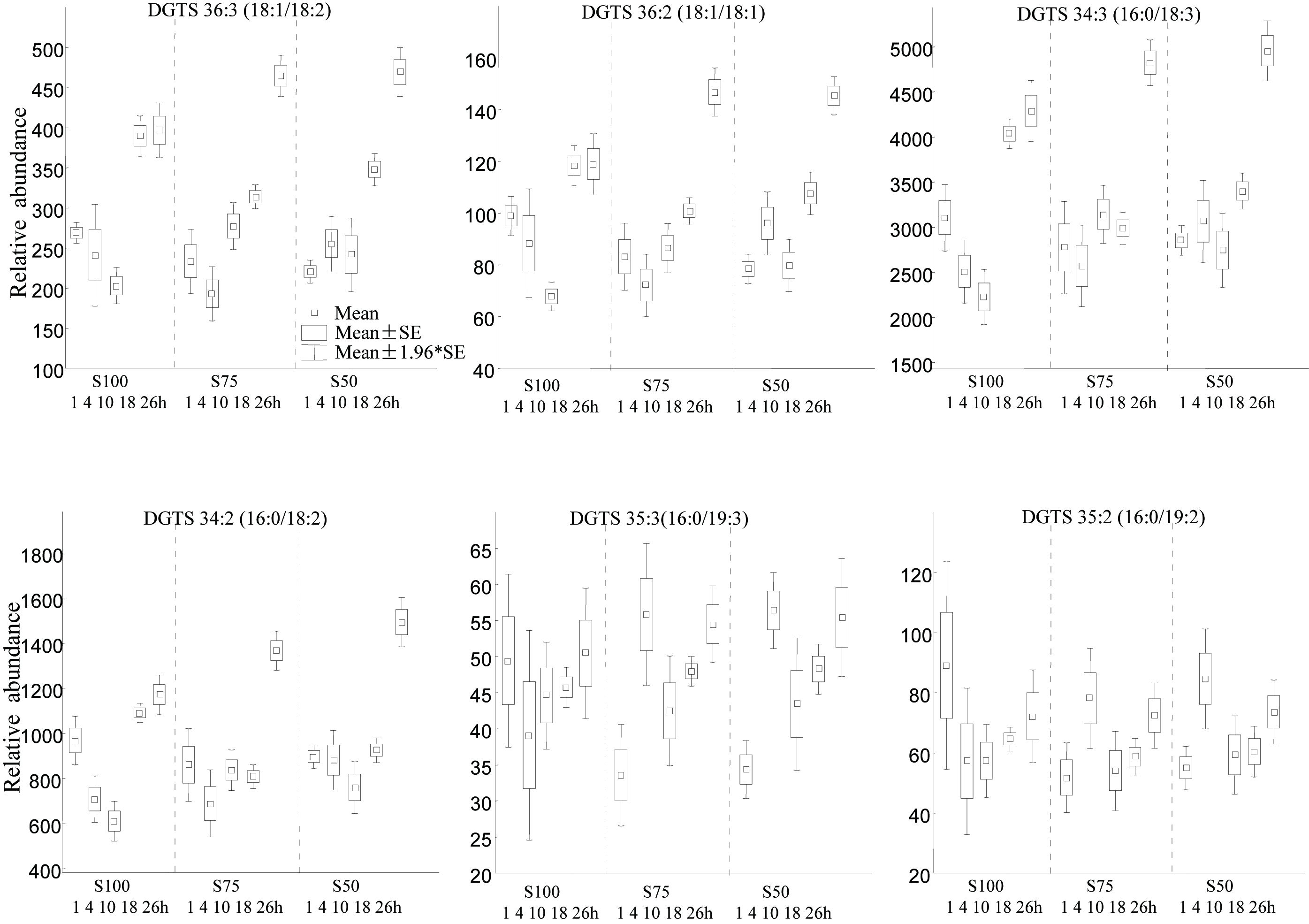

Supplement: S5 Fig — Arithmetic mean values with ±S.E. as box and ±1.96 S.E. as whiskers. (TIF) [file pone.0137948.s006.tif]
